# The Exclusion of the New York Delegates from the American Medical Association

**Published:** 1882-06

**Authors:** 


### EDITORIAL DEPARTMENT.

The Exclusion of the New York Delegates from the American Medical Association.—As we go to press the telegraph brings the news that the delegation from the New York State Medical Society has been denied admission to the annual convention of the American Medical Association in session at St. Paul, Minn. This result was not entirely unexpected, although it was hoped that the general clamor of the medical journalists throughout the country would not lead the Judicial Council of the Association to recommend this step. As an act of folly this action has no precedent in former proceedings of the American Medical Association. The attempt to discipline the New York State Society, which in age and the respectability of its membership is at least the equal of the National Association, by cutting off all official relations between the two, will perhaps be found a more serious matter for the latter than for the former. As we said in our last issue : "It is absurd to suppose that breaking off official relations with the most eminent medical men in the country would in any degree limit the influence which, as medical teachers, they exert upon medical opinion." We look forward with confidence to an early acceptance of the position occupied by our State Society, by similar organizations in all progressive sections of the country. The New York State Society has taken a stand on the side of progress, liberality, and humanity from which it should not recede. If it maintains its stand with firmness, the American Medical Association will eventually come over to the same side. The spirit of enlightenment, progress, and liberality is gaining ground even within its ranks, and in a very few years we may expect the repeal of the present antiquated—and in many of its articles—absurd code, and the adoption of one more nearly in accordance with the demands of the age.

